# Enhancing MRI contrast between sub-chronic myocardial infarct, remote and healthy myocardium using periodic irradiation in the rotating frames

**DOI:** 10.1038/s41598-025-34949-6

**Published:** 2026-01-06

**Authors:** Elias Ylä-Herttuala, Iida Räty, Ahmed Montaser, Svetlana Laidinen, Shalom Michaeli, Timo Liimatainen

**Affiliations:** 1 A.I. Virtanen Institute, University of Eastern Finland, Kuopio, Finland; 2 Clinical Radiology Unit, Imaging Center , Kuopio University Hospital , Kuopio, Finland; 3 Department of Pharmacy , University of Eastern Finland , Kuopio , Finland; 4 Center for Magnetic Resonance research , University of Minnesota , Minneapolis, MN USA; 5 Research Unit of Health Sciences and Technology University of Oulu , Oulu, Finland; 6 Department of Diagnostic Radiology , Oulu University Hospital , Oulu, Finland

**Keywords:** Cardiology, Medical research

## Abstract

Relaxations Along a Fictitious Field in the rotating frame of rank 2 (RAFF2) was used to characterize fibrosis-related myocardial diseases with MRI. RAFF2 can be sensitized to molecular dynamics processes by changing the durations of the RAFF2 pulses. Here, we studied the effect of altering the RAFF2 pulse durations for characterization of myocardial infarction (MI). 10 mice underwent myocardial infarct surgery, and the hearts were collected 7 days after the operation. 3 mice hearts were served as healthy control. We tested the influence of stretching factor (TL) on relaxations, where TL was between 0.6 and 2.0, and TL = 1 corresponds to the duration of the RAFF2 pulses TL = 2.25 ms with α_2_ = 45°. Here, α_2_ = 45° indicates location of the fictitious field in the second rotating frame. The contrast between MI and remote areas were measured as Relative Relaxation Time Difference (RRTD). Significant increase in relaxation time constants between MI and remote areas with relatively high RRTD were found in RAFF2 TL0.6, TL0.8, TL1.8 and TL2.0. With protein analysis, fibronectin was upregulated in MI area and histology showed area of fibrosis. By altering durations RAFF2 pulse, MI areas can be captured from the myocardium, which provides a novel way to evaluate molecular dynamics in MI area. The findings were validated by the results of other MRI methods, protein analyses and histological sections. Enhancement of MRI contrast between MI, remote and healthy myocardium without using any contrast agents can be achieved by changing the duration RAFF2 pulse.

## Introduction

**Fig. 1:**
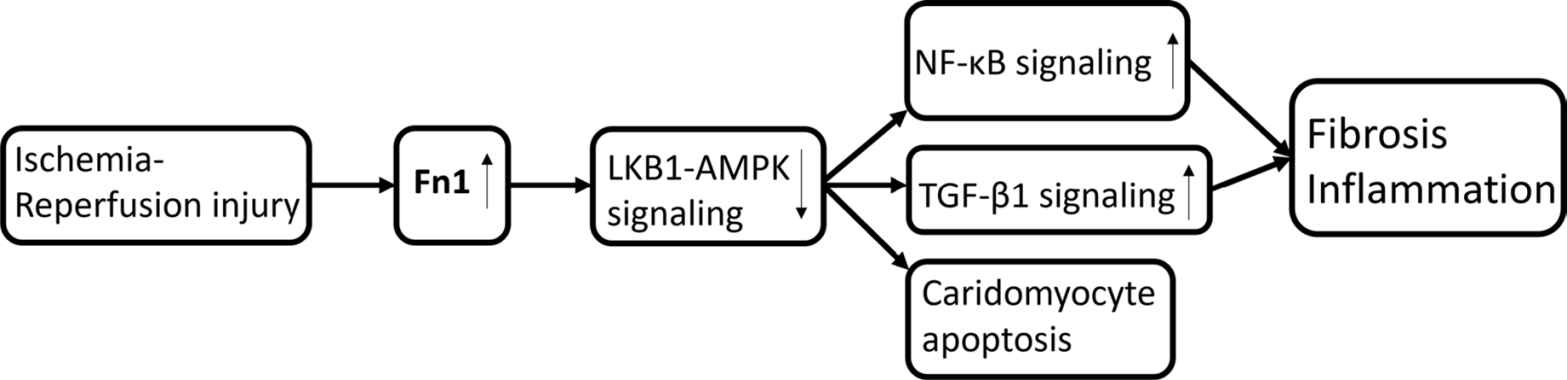
A schematic diagram of the ischemia-reperfusion injury, which is causing fibronectin (Fn1) to upregulate. This is causing the inhibition of liver-kinase B1 AMP-activated protein kinase (LKB1-AMPK) signaling, which is further promoting nuclear factor kappa-light-chain-enhancer of activated B cells (NF-KB) and transforming growth factor beta 1 (TGF-B1) signaling pathways leading to cardiomyocyte apoptosis, fibrosis and inflammation. The figure is modified from^2^.

Occlusion of coronary artery leads to oxygen deprivation, myocyte loss and subsequently to scar formation^1^. The development of myocardial infarction (MI) after the occlusion of left anterior descending artery (LAD) affects biological processes, such as protein up- and downregulation and furthermore in the protein content (Fig. 1). Previous studies have shown that fibronectin (Fn1) plays a critical role in the development of MI since it is a component of the extracellular matrix (ECM)^2^. Fn1 is involved in cellular adhesion, growth, angiogenesis, the formation of the fibronectin matrix and collagen fibrils within the damaged myocardium since polymerization of Fn1 is required for collagen deposition^2^. Thus, it contributes to the remodeling, and to fibrosis of the myocardium after the MI (Fig. 1)^2^. There are also several proteins which are involved in the development of MI, such as tenascin-C, osteopontin, periostin (postn), and thrombospondins^3^.

Conventionally, in vivo MI is determined with late gadolinium enhancement (LGE) magnetic resonance imaging (MRI), which is based on the differences in wash-out rates of gadolinium from MI and remote tissues^4,5^. However, LGE has major drawbacks, since the hyperintensity areas are generated only by the expansion of the extracellular volume (ECV). Gadolinium wash-out similarly from areas with scar, fibrosis and mild collagen formation^2^, making LGE incapable of distinguishing these biological processes behind different cardiovascular diseases^2^. Other drawbacks are related to invasiveness, time consumption, and toxicity for using LGE^6^.

Conventional T_1_ and T_2_ relaxation time mapping methods can be used to distinguish the MI area from the remote and healthy myocardium^4,7^. T_1_ relaxation time constant is sensitive to MI and fibrotic areas, which is seen as enhanced T_1_ relaxation time constant in that area, whereas T_2_ relaxation time constant is sensitive to edema caused by ischemic and inflammation reactions in the MI area, which is seen as enhanced T_2_ relaxation time constant^4,5^. Despite the success of T_1_ and T_2_ mapping methods for determining MI areas, these methods have some limitations, such as limited robustness for cardiac fibrosis determination by T_1_ and lack of specificity in the relaxation dispersion of the T_2_ measurements^8^. Diffusion tensor imaging is also one promising MRI method to delineate MI area from remote area^9^.

One MRI contrast, which has been tested in humans is a longitudinal rotating frame relaxation, is characterized by T_1ρ_ relaxation time constant. T_1ρ_ occurs when the magnetization is aligned along the applied radiofrequency (RF) pulse effective magnetic field. T_1ρ_ measurements can be performed with either a continuous wave (CW) spin-lock (SL) technique with the SL pulses at the constant amplitude^10,11^, or with a train of adiabatic pulses placed prior to the readout portion of the sequence^12–15^. CW T_1ρ_ is increased in MI compared to remote areas, which have been demonstrated earlier in mice^16–18^, in swine^10^ and in humans^19^. Additionally, a good correlation between CW T_1ρ_ relaxation time constant maps and LGE based determination of MI area was found in mouse^16^ and swine in vivo models^10^. Adiabatic rotating frame measurements can be modulated with the choice of frequency- and amplitude-modulation functions of adiabatic pulses, which allows them to generate tissue contrast non-invasively and tamper the contrast in the myocardium after MI^16^. Adiabatic T_1ρ_ relaxation measurements have been used to image MI area in mice in vivo and ex vivo hearts where adiabatic T_1ρ_ relaxation time constants were elevated in myocardial injury regions compared to remote areas^20,21^.

In addition to T_1ρ_, magnetization can be aligned perpendicular to the effective field. Then, magnetization decays with transversal rotating frame relaxation time constant T_2ρ_^7,22^. Adiabatic T_2ρ_ has been used successfully to determine MI area from the rest of the myocardium in acute and subchronic phases of MI in both ex vivo and in vivo mice^21^ indicating greater potential of adiabatic T_1ρ_ and T_2ρ_ measurements to assess the specific pathological processes, such as fibrosis, during the development of the MI area as compared to conventional free-precession T_2_ method.

At high magnetic field, adiabatic T_1ρ_ and T_2ρ_ relaxation mapping methods are limited by specific absorption rate (SAR)^16^. One alternative method is entitled Relaxation Along a Fictitious Field in the rotating frame of rank n (RAFFn). Magnetization is swept under sub-adiabatic conditions during RAFFn which significantly reduces power deposition without compromising final effective field amplitude. The delivered specific absorption rate is significantly lower compared to T_1ρ_ and T_2ρ_^16,23,24^. Image contrast enhancement based on periodic RF irradiation has been under research interest for a long time^25–31^. By varying the period of refocusing of the rotating frame rotary echoes generated during RAFFn, the sensitivity of RAFFn to exchange-induced relaxations can be tuned with periodic RF irradiation to the differences in chemical shifts between the exchanging sites^21^. Notably, RAFFn (n is rank of the rotating frame) was designed to operate in different rotating frames (e.g., *n* = 1–5), and the RAFF2 (rank = 2) was used in cardiac MRI applications^16,17,24,25,32^. For example, it was demonstrated in several prior in vivo studies that RAFF2 can successfully characterize fibrosis-related myocardial diseases^16,17,25^, with a good correlation to LGE^16^, it owes potential to be further tuned to increase contrast between MI and remote myocardium by altering the refocusing period of the RAFF2 pulses.

In this work, we measured and localized MI area in mouse ex vivo hearts using RAFFn to study the effect of the refocusing period on RAFF2 relaxation time constants. In addition, single protein up- or downregulation effects on RAFFn relaxations were studied.

## Results

### Increased relaxation times in remote areas compared to healthy hearts

**Fig. 2:**
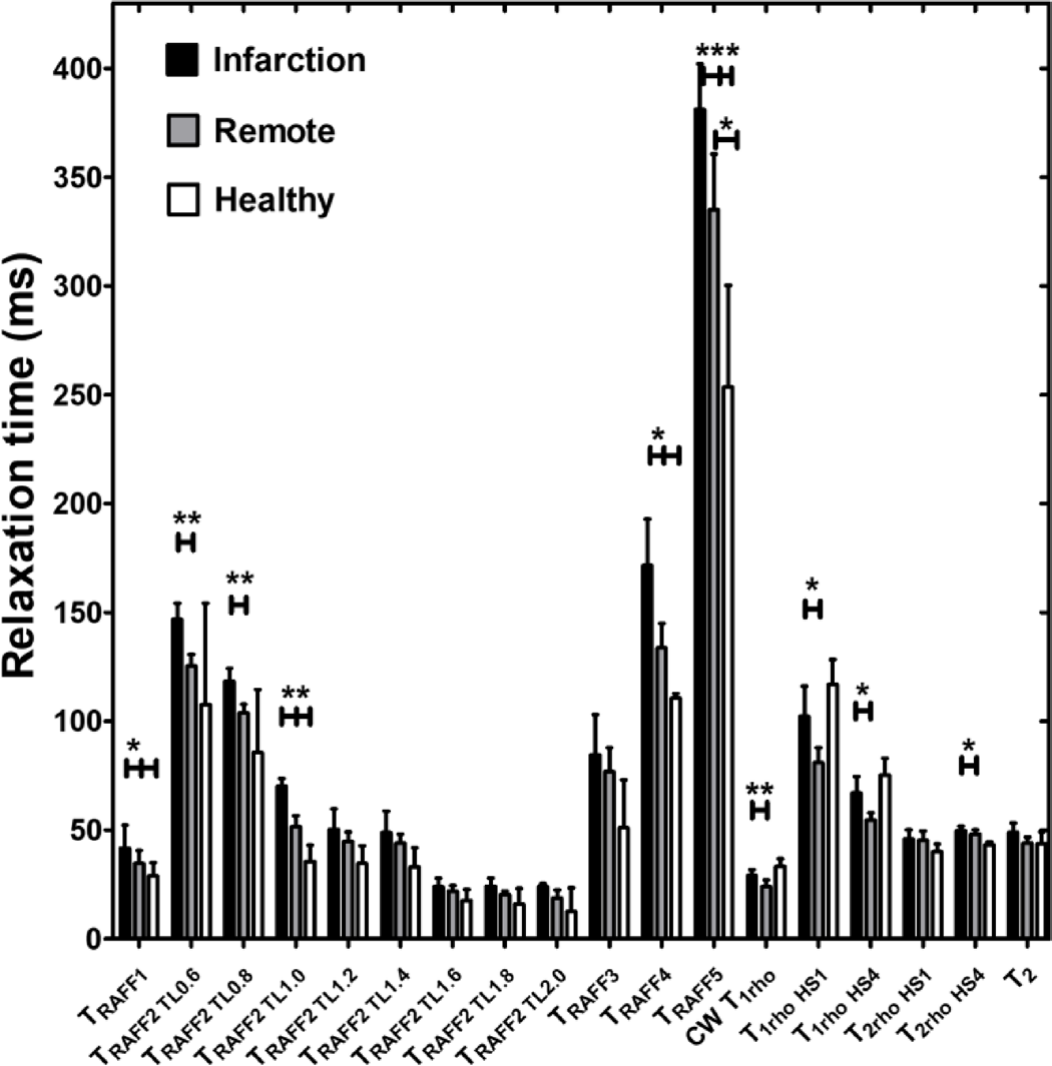
Averaged rotating frame relaxation times of 10 infarcted hearts and 3 healthy control hearts used in ex vivo measurements. Statistical significances were tested with Two-way ANOVA with Bonferroni post hoc correction test (*P < 0.05, **P < 0.005 ***P < 0.001).

Relaxation time constants RAFF1 (*p* < 0.05), RAFF2 TL1.0 (*p* < 0.01), RAFF4 (*p* < 0.05) and RAFF5 (*p* < 0.05) were significantly higher in remote areas compared to healthy hearts (Fig. 2). Other methods were unable to detect the difference (Fig. 2). Significant differences between MI and healthy control hearts were found in RAFF1-5 (*p* < 0.05) relaxation time constants (Fig. 2).

### Increased relaxation times in MI compared to remote areas

**Fig. 3:**
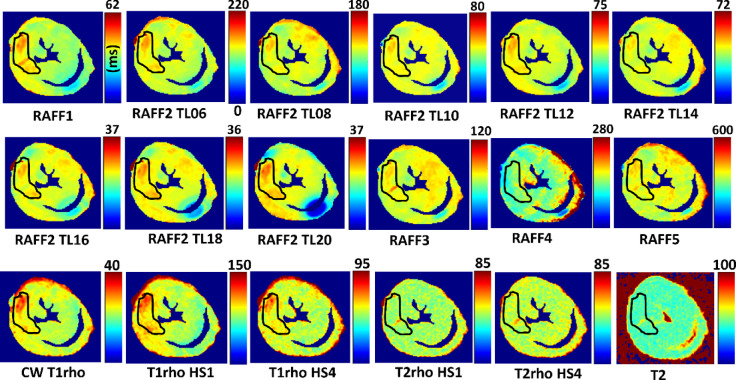
Example of rotating frame and T_2_ relaxation time constant maps in one infarcted heart. Black ROI outline indicates the area of MI, and the remote area was defined in the septum, which was selected in RAFF2 TL1.0 relaxation map and subsequently this MI area was used for the analysis in all other relaxation maps.

**Fig. 4:**
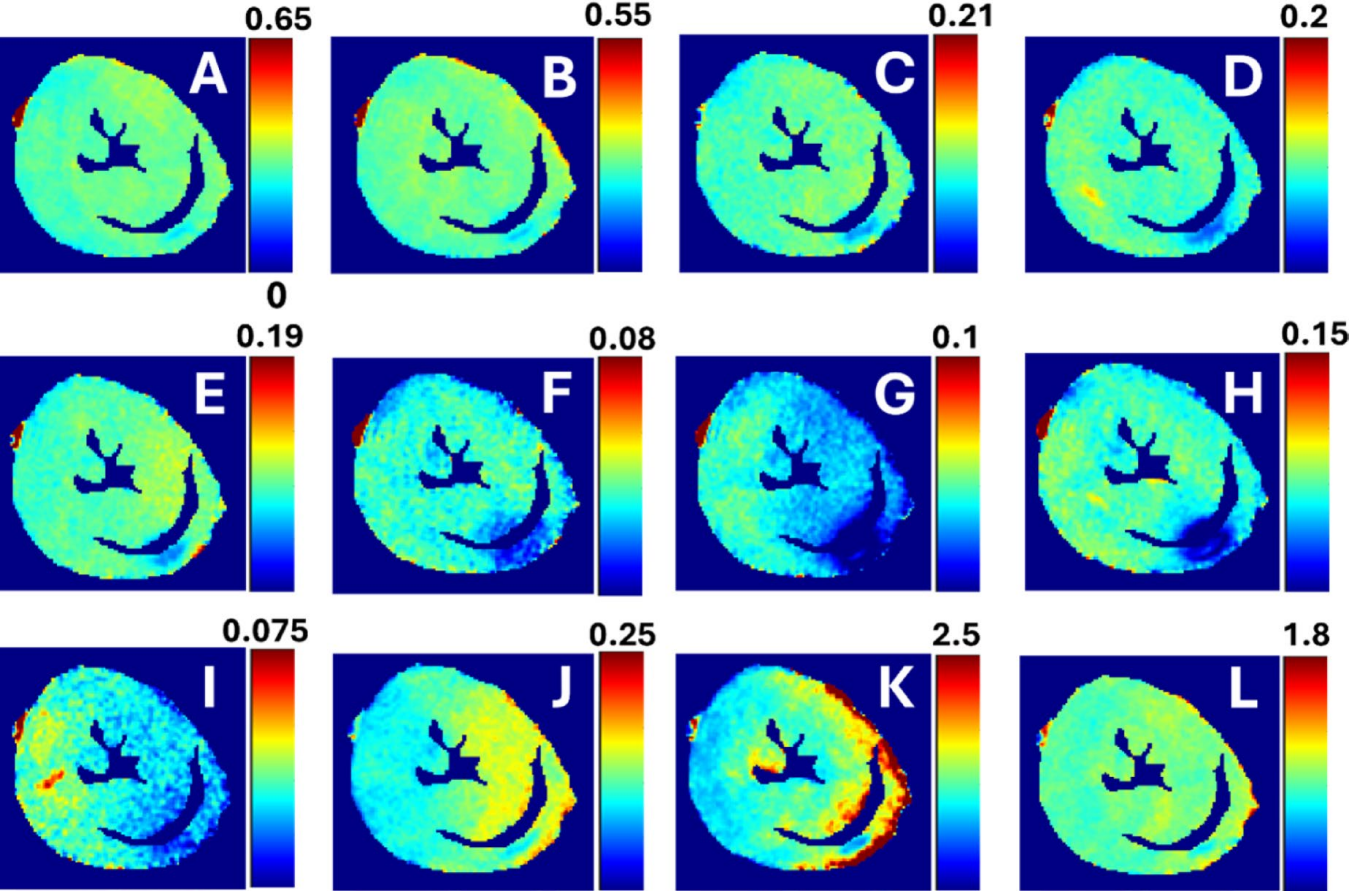
Example of SS-maps from the same heart as in Fig. 3. There are SS-maps from RAFF2 TL 0.6 (**A**), RAFF2 TL0.8 (**B**), RAFF2 TL1.0 (**C**), RAFF2 TL1.2 (**D**), RAFF2 TL1.4 (**E**), RAFF2 TL1.6 (**F**), RAFF2 TL1.8 (**G**), RAFF2 TL2.0 (**H**), RAFF1 (**I**), RAFF3 (**J**), RAFF4 (**K**) and RAFF5 (**L**).

In all calculated relaxation maps, higher relaxation time constants are associated with MI as compared to the remote myocardium (Figs. 2 and 3). Calculated steady state (SS)-maps from different RAFFn methods are presented in Fig. 4. The effect of periodicity of irradiation by increasing the TLs of *P*-packets from 0.6 to 2.0, where TL = 1 corresponds to the duration 2.25 ms can be seen as monotonic decrease in the RAFF2 in MI, remote area and healthy control hearts by being the highest in the TL0.6 and the lowest in TL2.0 (Fig. 2). Furthermore, significantly increased relaxation time constants were found in RAFF2 TL0.6 (*p* < 0.01), RAFF2 TL0.8 (*p* < 0.01) and RAFF2 TL1.0 (*p* < 0.01) maps in the MI area as compared to remote myocardium (Fig. 2). As a comparison to RAFF2 results, significant increase in relaxation time constants were found with RAFF1,4,5 (*p* < 0.05), T_1ρ_ (*p* < 0.05) and T_2ρ_ (*p* < 0.05) in the MI area as compared to remote myocardium (Fig. 2).

### RAFF2 TL2.0, RAFF2 TL1.8 and RAFF1 exhibited the highest contrast between MI and remote areas as described by RRTD values

**Fig. 5:**
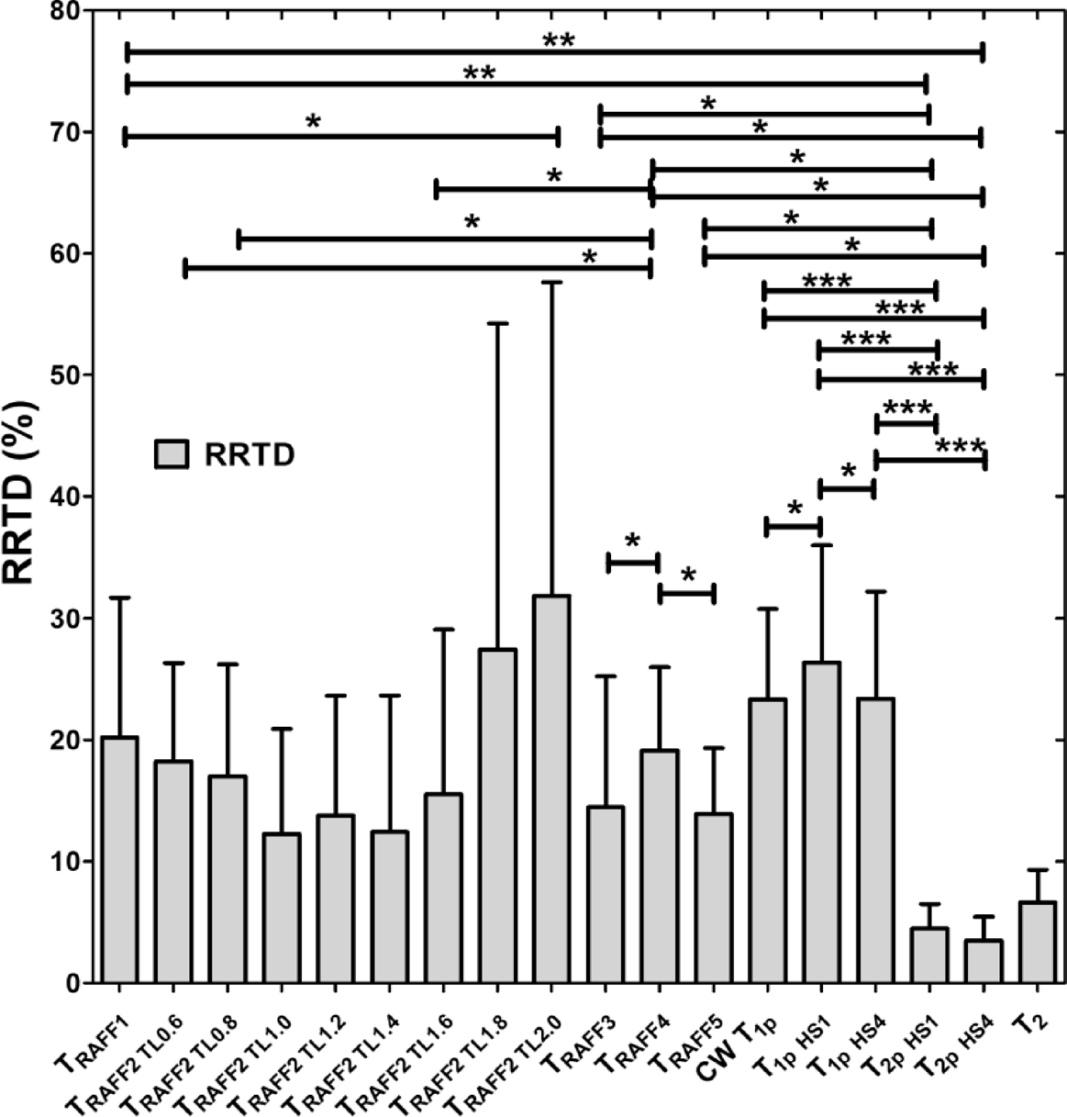
RRTD values calculated from relaxation times between MI and remote areas. The statistical comparisons were calculated between methods with Students’ T-Test and One-way ANOVA Dunn’s multiple comparison test (* *p* < 0.05, *** *p* < 0.001).

The contrast difference between MI and remote areas was studied visually and with relative relaxation time difference (RRTD)-values (Figs. 3, 4 and 5). The highest RRTD values were seen with RAFF2 TL2.0, RAFF2 TL1.8 and RAFF1 methods (Fig. 5). By shortening the *P*-packets as they had significant difference between MI and remote areas, the RRTD values were in RAFF2 TL0.6 (18.2 ± 0.8%), in RAFF2 TL0.8 (17.0 ± 0.9%), and in RAFF2 TL1.0 (12.3 ± 0.8%). Significant differences were also found between RAFFn and different RAFF2 TL RRTD values (Fig. 5). However, no significant differences in RRTD values between longer *P*-packets (TL1.4-TL2.0) were found, but there were contrast differences between the MI and remote areas as demonstrated in Fig. 3.

For comparison to RAFFn measurements, the RRTD values from all T_1ρ_ methods were lower than RRTD values of RAFF2 TL2.0 and RAFF2 TL1.8 methods and higher than RAFF1 RRTD values (Fig. 5). Additionally, significant differences in RRTD values between T_1ρ_ (hyperbolic secant) HS1- T_1ρ_ HS4 (*p* < 0.05), and CW T_1ρ_ - T_1ρ_ HS1 (*p* < 0.05) were found (Fig. 5). For the other comparison to RAFFn and all T_1ρ_ to T_2ρ_ measurements, significant differences in RRTD values were found between RAFF3,4 - T_2ρ_ (*p* < 0.05), RAFF5 - T_2ρ_ (*p* < 0.01), all T_1ρ_ - T_2ρ_ methods (*p* < 0.001) (Fig. 4). Comparing the rotating frame methods to T_2_ method, there were no significant differences found (Fig. 5). B_1_ measurements resulted in 620 ± 11 Hz.

### Simulated RAFF2 TL 0.6–2.0.6.0 and RAFFn relaxation times were not affected by single protein up or down regulation

**Fig. 6:**
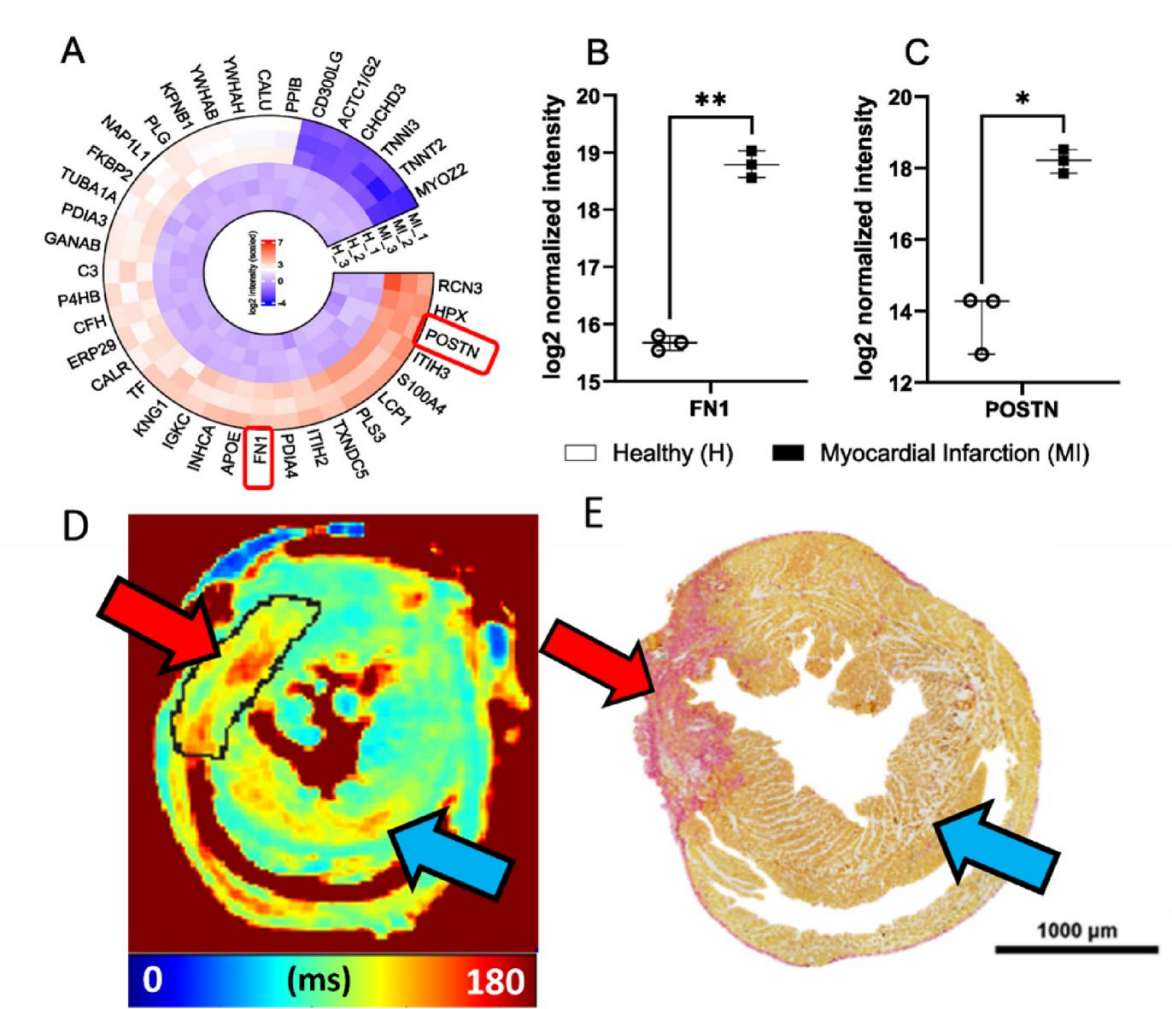
(**A**) Top 40 proteins which are statistically significant (Adjusted p value < 0.05, logFC > 2) and differentially expressed in MI mouse model (MI15, MI16, MI18) compared to healthy wildtype littermates (H19, H20, H21). Red colour is showing the upregulation and the blue the downregulation. (**B**) Fn1 and in (**C**) Postn intensity in the MI and healthy myocardia. (**D**) RAFF2 relaxation time map 7 days after the MI. (**E**) Sirius Red histology section from one heart, where red area indicates the area of fibrosis and MI. Red arrows are pointing the area of MI and blue arrows are pointing the remote myocardium.

The top 40 proteins were found when the protein analysis of MI and healthy myocardium were done (Fig. 6). There were multiple proteins, which were downregulated in healthy myocardium and upregulated in MI area, like fibronectin (Fn1), Reticulocalbin-3 (Rcn3), Hemopexin (Hpx), Isoform 2 of Periostin (Postn), Inter-alpha-trypsin inhibitor heavy chain H3 (Itih3) and Protein S100-A4 (S100a4) proteins (Fig. 6). On the other hand, for example, Myozenin-2 (Myoz2), Troponin T cardiac muscle (Tnnt2), Troponin I cardiac muscle (Tnni3), Myosin light chain 1/3 skeletal muscle isoforms (Myl; Myl3) and Actin, gamma-enteric smooth (Actc1/Actg2) proteins were upregulated in healthy myocardium but downregulated in MI tissue (Fig. 6). The most important finding was that Fn1 protein (approximately 8.9-fold) and Postn protein (approximately 9.0-fold) were significantly upregulated in the MI area compared to healthy myocardium supporting knowledge of the development of the MI (Fig. 6).

Results of simulations showed that the concentrations of Fn1 and Postn proteins are too low to affect the relaxation time constants and SS-values of different RAFFn and RAFF2 methods after the MI (Table 2). The SS-values in MI area were higher for different RAFF2 TL’s and similar with the original RAFF1-4 in simulations as compared to SS-values from the measurements (Table 2; Figs. 2 and 4). Additionally, the results of simulations are in line with measured relaxation time constants since RAFF2 TL0.6 and RAFF2 TL0.8 have smaller R-values as compared to bigger R-values in RAFF2 TL1.0–TL2.0 methods (Table 2; Figs. 2 and 4).

### Histology revealed the MI area with elevated fibrotic collagen content

Picro Sirius Red-stained histology revealed the MI area with elevated collagen content and fibrosis in the myocardium as compared to the rest of the myocardium (Fig. 6). Small, delicate stained areas were also seen in the remote areas and the original RAFF2 relaxation time map showed the increase of RAFF2 relaxation time constant in the same remote areas as in the Picro Sirius Red stained section (Fig. 6). MRI imaging slices were acquired in the same location as the histology slices were cut, which supports our MRI findings. In addition, as the protein analysis was done by cutting the hearts in half, protein analysis in the MI area included also some parts of remote areas of the myocardium, which can be seen in the histology (Fig. 6). These observations are supporting also the protein findings.

## Discussion

In this study, the determination of MI, remote and healthy areas were done by either compressing or stretching the *P*-packets in RAFF2 pulse train. As a comparison, other rotating frames and T_2_ relaxation time constants were determined in the same areas as with the RAFFn method and the change of *P*-packets in RAFF2 pulse. Additionally, MS proteomics analysis was done for the determination of MI and Picro Sirius Red-stained histology validated the MI area.

### Increased relaxation times in remote areas as compared to healthy hearts

We found that relaxation time constants in RAFF1, RAFF2 TL1.0, RAFF4 and RAFF5 maps differed significantly between remote areas and healthy hearts. This is interesting, since it is known that remote areas are affected by stress, edema and inflammation reactions. This is supported by the earlier study, where it was found that CW T_1ρ_ relaxation time constant increased in the remote myocardium as compared to healthy myocardium; however, the difference between those regions were not significant^18^. Histology revealed tissue changes detected by a delicate Picro Sirius Red staining in the remote areas as compared to healthy myocardium (Fig. 6). In addition, original RAFF2 relaxation time map showed the increase of RAFF2 relaxation time constant in the same remote areas as in the Picro Sirius Red stained section (Fig. 6).

### Increased RAFF2 and other rotating frame relaxation time constants in MI compared to remote areas

MI area had increased rotating frame relaxation time constants in RAFF1, RAFF2 TL0.6, RAFF2 TL0.8, RAFF2 TL1.0, RAFF4, RAFF5, CW T_1ρ_, adiabatic T_1ρ_, and adiabatic T_2ρ_ relaxation maps as compared to relaxation times in the remote area (Figs. 3 and 4), which agrees with previous studies^10,16,20,21^ and supports the idea of RAFF2 TL0.6-TL2-0 together with other rotating frame methods being a contrast agent free method to separate the MI area from the rest of the myocardium. Relaxation time constants measured with RAFF2 TL0.6, RAFF2 TL0.8 and RAFF2 TL1.0 relaxation times increased significantly in MI area as compared with remote areas, which indicates that reducing the durations of *P*-packets in RAFF2 pulse train affect the relaxation time constants in MI area while keeping the contrast difference between MI and remote areas (Figs. 3 and 4). The durations of *P*-packets affect the time to repeat the *P*-packets in the preparations to acquire different weighting images, which could have potential to add the sensitivity to reveal the MI area from the rest of the myocardium. Furthermore, elevated relaxation time constants were found in the same MI areas as determined with RAFFn, T_1ρ_ and T_2ρ_, indicating the benefits of the shortening the *P*-packets in RAFF2 pulse.

### RAFF2 TL2.0, RAFF2 TL1.8 and RAFF1 exhibited the highest contrast between MI and remote areas as described by RRTD values

The highest contrast differences between MI and remote areas described by RRTD values were RAFF2 TL2.0, RAFF2 TL1.8 and RAFF1 methods, while mean RRTD values in RAFF2 TL0.6, in RAFF2 TL0.8, and in RAFF2 TL1.0 were higher than 10% indicating the contrast differences between MI and remote areas. The highest RRTD among the methods was RAFF2 TL2.0. With shortening the *P*-packets in RAFF2 pulse, the relaxation time constants had a significant difference between MI and remote areas; however, those methods did not have as high RRTD-values as stretching the *P*-packets but still RAFF2 TL0.6, in RAFF2 TL0.8, and in RAFF2 TL1.0 had high RRTD-values. These findings can be interpreted by the effect of chemical exchange since exchange-induced relaxation rate constants causing regional change of relaxation time constants, especially in MI area, where macromolecular tissues like collagen, fibrosis and scar are present. As compared to the original RAFF2, which had the smallest RRTD-value, by changing the length of *P*-packets in RAFF2 pulse can increase the RRTD between MI and remote areas.

Interestingly, T_1ρ_ with HS1 had the largest RRTD value between the MI and remote areas when compared to CW T_1ρ_ and T_1ρ_ with HS4 methods. The substantial differences observed in T_1ρ_ with HS1 pulse in comparison with other T_1ρ_ techniques may be related to the specific motional regime selected by the adiabatic T_1ρ_ approach utilizing HS1 pulse modulation, which was also seen in previous study^20,21^. RRTD values of T_1ρ_ significantly differed from T_2ρ_, which is attributed to the differences in relaxation channels which govern T_1ρ_ and T_2ρ_ relaxations. We also found significant difference in RRTD values between RAFFn and T_2ρ_, which supports the findings of the rotating frame relaxation time constant differences between MI and remote areas. This is interesting since it has been shown before that T_RAFFn_ comprises both T_1ρ_and T_2ρ_ relaxation pathways^23^ but we did not see any significant differences between RAFFn and T_1ρ_, which might indicate that the increase of relaxation time constant in MI area is due to similarity of the relaxation mechanisms and the biological tissues, such as collagen and fibrosis^20^.

Comparing the rotating frame methods to T_2_, there were no statistically significant differences in RRTD values between these methods even though the relaxation pathways are different between these methods. It should be noted that we have done ex vivo measurements, where the relaxation mechanisms are affected due to formalin fixation, since protein and water molecule interactions in ex vivo and in vivo MRI could substantially differ in their spin dynamics, which leads to almost uniform fixation of the ECM and causes heart dehydration leading to alterations in proton diffusion patterns as compared to in vivo situation^33^. Additionally, in ex vivo MRI, the maximum temperature of the heart is room temperature, which is different than during in vivo measurements and needs to be taken account^34^.

### RAFF2 TL 0.6–2.0.6.0 and RAFFn were not affected by single protein up or down regulation

Proteomics analysis revealed that cell adhesion proteins like Postn and Fn1 are related to outside of myocyte in ECM, fibronectin matrix, fibril formation and cardiac repairing (by Postn protein) and these proteins were highly upregulated in MI area in our results and previous findings^2,35^. Additionally, other upregulated proteins in MI area like Itih3 and S100a4 are related to other ECM proteins, calcium-binding, angiogenesis, and various cellular processes, indicating that there have been tissue changes in the ECM as compared to healthy myocardium by determining the changes in protein content in MI area. Fn1 protein is interesting because it is related to ECM, fibronectin matrix and fibril formation since polymerization of Fn1 is required for collagen deposition and thus, it plays a critical part in fibrosis in MI area^2^.

The gold standard in MI visualization with MRI is LGE, which is related to Gd’s wash-in-wash-out-rate from MI area ECM compared to remote area ECM. In addition, relaxation time constants are increasing due to the increase of free water in MI area, which is increasing the ECV and ECM. Thus, protein findings in this study support the idea of rotating frame relaxation time methods to efficiently determine the MI area from the remote areas, which is successfully shown in previous MI studies^10,16,19–21^. Furthermore, Fn1 has been previously analyzed with nuclear magnetic resonance^36^, indicating that Fn1 has enough MR properties to be able to get detectable MR-signal from Fn1; however, further studies of how Fn1 itself influences the detectable MRI-signal. For the detectability of Fn1, the actual concentration of Fn1 in MI area, how the pH is changing during the MI development and how the free water molecules can access the exchange site of Fn1 need to be figured out to consider Fn1 role in the effect to the relaxation. In addition to protein findings, there have been studies about cardiac ECM target imaging for the fibrotic collagen content with MRI^37,38^ and with collagen targeted contrast agent^39^, which indicate that more specific MRI methods can be optimized to sensitively capture the ECM related proteins in the MI area.

Our simulations of the relaxation time constants and SS formation for Fn1 and Postn protein upregulations in MI area showed that the concentrations of these proteins are too low to affect the RAFF2 TL and RAFFn relaxation times or steady states, even though Fn1 can produce the detectable NMR-signal^36^. Straightforward connection between Fn1 and the increase of rotating frame relaxation times cannot be made based on our simulations; however, the sum of Fn1 and other upregulated ECM proteins have a minor effect on detectable MR-signal. Based on our histology results, the major source of detectable MR-signal is from increased fibrosis and water accumulation, which might overshadow the MR-signal from the upregulated ECM proteins in heart imaging. In addition to that, there were also differences in remote and healthy myocardium detected with the RAFFn method. Here, one possible factor in that result is related to the role of Fn1 and Postn proteins in whole MI differences compared to healthy myocardium.

## Limitations

There were some limitations in this 2-D ex vivo study. The sample size was small, particularly healthy hearts, which affected the robustness of the statistical analysis both MRI and MS proteomics. Exact location of 2-D short-axis MRI slice might not always be optimal as the more serious MI area might have been lower in the myocardium. Furthermore, the relaxation times measured with RAFF2 with increased TL were more prone to were B_0_ artifacts than RAFF2 TL1.0 (Fig. 4). As original RAFF1-5 pulses have been applied for human brain imaging and RAFF2 for human heart imaging, due to similarities of different lengths of RAFF2 pulses to original RAFF1-5 pulses, this method owes potential to be transferred to in vivo preclinical and clinical devices. However, the results of this study may not be transferrable as is, due to differences between in vivo and ex vivo tissue conditions. To the best of our knowledge, this study is the first demonstration of a novel contrast in cardiac MRI which is generated during periodic irradiation.

As a conclusion, RAFF2 rotating frame relaxation contrast between infarct and remote myocardium depends on and can be optimized by altering the duration of refocusing time. RAFFn were able to distinguish MI, remote and healthy myocardia without a need of contrast agent.

## Methods

### Animals

The hearts of C57BL mice (*n* = 13) were obtained from the Animal Center of the University of Eastern Finland. The hearts from 10 mice were collected after 7 days of Left Anterior Descending LAD occlusion and 3 hearts were from healthy controls and no sham operations were done to healthy controls. Animal experiments were performed according to the national and international guidelines for laboratory animal use, and the experiments were approved by the National Animal Experiment Board of Finland under license ESAVI-270-04.10.10.07–2017 and carried out following the guidelines of the Finnish Act on Animal Experimentation and Directive 2010/63/EU of the European Parliament. All experiments were following the Animal Research Reporting In Vivo Experiments (ARRIVE) guidelines.

LAD was ligated in a surgical operation^40^, where mice were the first anesthetized with inhalation anesthesia (4% induction, 2% maintenance with 30/70 oxygen/nitrogen ratio). Mouse chest was opened at the 4th intercostal space of the sternum. After that, the heart was pulled out, pericardial sac was removed and then LAD occlusion was done. The heart was then returned to its initial place and the chest was closed. After the operation, analgesia was given subcutaneously to mice on the day of the operation and the following three days. The analgesia included buprenorphine (0.3 mg/ml), Temgesic (0.05–0.1 mg/kg), Carprofen (50 mg/ml) and Rimadyl (5 mg/kg). After 7 days, mice were sacrificed with CO_2_ and their hearts perfused with phosphate buffer saline through the left ventricle. Then the hearts were collected and fixed with 4% paraformaldehyde in 7.5% sucrose for 4 h and after that, the hearts were stored in 15% sucrose.

### MRI

MRI was performed with a vertical 9.4 T magnet using volume RF transceiver coil with diameter of 15 mm (Rapid Biomedical GmbH, Rimpar, Germany) and Vnmrj3.1 Varian/Agilent DirectDrive console. All imaging was conducted ex vivo. The hearts were placed in 8-mm NMR glass tube, and the tube were filled with perfluoropolyether (Galden HS 240, Solvay Solexis, Italy), since Galden is a fluorinated heat transfer fluid, which is not giving any MR signal.

**Fig. 7:**
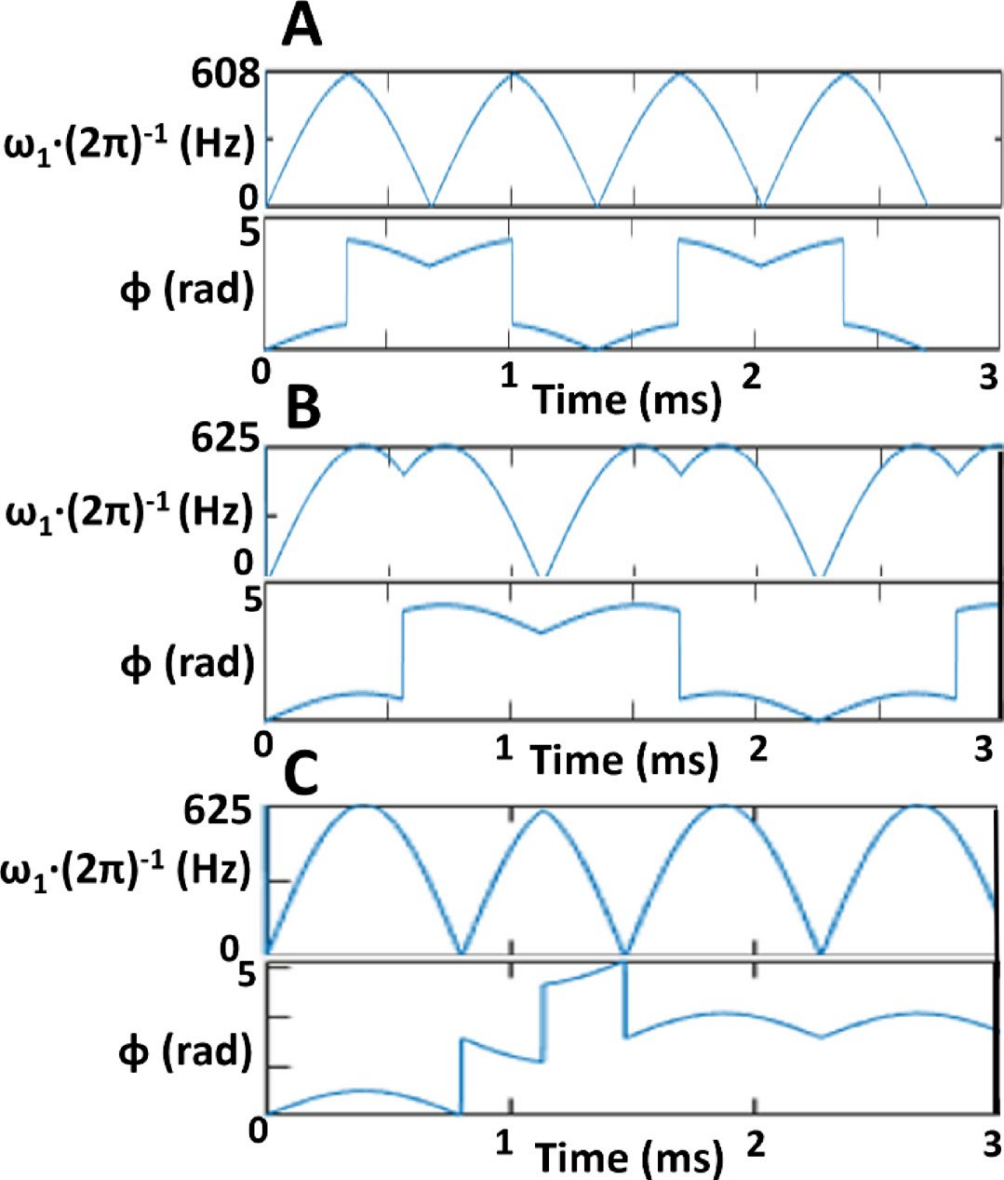
Effect of the stretching factor on the amplitude of *P*-packets and to the phases in RAFF2 pulse. Here, the RAFF2 TL0.6 (**A**), RAFF2 TL1.0 (**B**), and RAFF2 TL2.0 (**C**) methods are shown.

RAFFn pulse wave forms were generated as before^23,26^ with α_1_ = 45° and refocusing durations were calculated with the equation of 4*π*/($\:\sqrt{2{{\upomega\:}}_{1}^{max}}$), where $\:{{\upomega\:}}_{1}^{max}\:$is maximum frequency of applied B_1_ as described in^23,26,41^. Imaging protocol contained experiments with RAFFn (*n* = 1–5), and RAFF2 with stretching factors (TL)s of 0.6–2.0.6.0, where TL0.6 is shortened by factor 0.6 from the original TL1.0 RAFF2 *P*-packet with the duration 2250 µs at the nominal peak power 625 Hz with α_2_ = 45°, where, α_2_ = 45° indicates location of the fictitious field in the second rotating frame, and TL2.0 is stretched by factor 2.0 (Fig. 7; Table 1) similarly as in^23,26,41^. The parameters of the pulses, i.e., the nominal pulse peak powers and the durations of *P*-packets with refocusing durations for all used RAFF2 pulses are presented in Table 1. The maximum number of pulses in the pulse train for the RAFF2 TL0.6-TL2.0 varied from 0 to 32 and the durations of the pulse trains were between 0 and 72.32 ms, respectively. The same pulse train lengths were applied for RAFFn as RAFF2 TL1.0 and the peak nominal pulse power ranged between 625 and 245 Hz, respectively^23^. Relaxation time constant maps for RAFFn measurements were calculated pixel-by-pixel manner with monoexponential decay function with SS-formation using Aedes Software (http://aedes.uef.fi) in MATLAB R2023b (Mathworks Inc., Natick, CA, USA). Signal intensity decay curves (*SI*(*t*)) were measured using two consecutive acquisitions, first starting with magnetization oriented along + *z*, and second when magnetization is initially along *-z*. Two SI curves were simultaneously fitted using non-linear fitting with a least square cost function using exponential decay and recovery to the SS^41^:

**Table 1:** Parameters of *P*-packets for different stretching factors in RAFF2 pulse.

RAFF2 TL	Peak amplitude of the pulse (Hz)	Length of *P*-packet (ms)	Length of refocusing (ms)
0.6	608	1.35	0.34
0.8	625	1.8	0.45
1.0	625	2.25	0.57
1.2	625	2.7	0.68
1.4	625	3.15	0.79
1.6	625	3.6	0.9
1.8	625	4.05	1.01
2.0	625	4.55	1.14

1 $${\mathrm{S}}{{\mathrm{I}}_{ \pm Z}}{\mathrm{=S}}{{\mathrm{I}}_{0, \pm Z}}{{\mathrm{e}}^{{\mathrm{-Rt}}}}{\mathrm{-}}{{\mathrm{S}}_{SS}}\left( {{\mathrm{1-}}{{\mathrm{e}}^{{\mathrm{-Rt}}}}} \right)$$

Here *S*_0_ is initial SI, *R* is the relaxation rate constant which describes the decay of the signal, *S*_SS_ is the steady-state SI for ${\mathrm{t}} \to \infty$SS is a fraction $SS={S_{SS}}/{\mathrm{S}}{{\mathrm{I}}_0}$

For comparison, CW T_1ρ_, adiabatic T_1ρ_ with both HS1 and HS4 pulses with *R* = 20^14^, adiabatic T_2ρ_ with both HS1 and HS4 pulses and *R* = 20, T_2_ and B_1_ were measured. For CW T_1ρ_ measurements, the flip of M to xy-plane was done with adiabatic half passage (AHP) pulse, which duration was 4.0 ms and the RF power was 2500 Hz^42^. After the flip, CW pulses with durations of 0, 18, 36 and 54 ms were used with pulse power of 1250 Hz to produce the CW SL weighting. After CW pulse, AHP reverse pulse was used to return M back to its initial orientation along the z axis. For both adiabatic T_1ρ_ and T_2ρ_ measurements, number of HS1 and HS4 pulses were 0, 8, 16, and 32 with duration of 4.5 ms and RF peak power of 2500 Hz in the pulse train^15,20,21^. For T_2ρ_ measurements, similar AHP and reverse pulses as in CW T_1ρ_ measurement was used before and after the adiabatic full passage (AFP) pulse train^15,21^. For T_2_ measurements, number 0, 4, 8 and 16 pulses with duration of 4.0 ms, delays of 0.55 ms and power of 2500 Hz were used to make the echo times of 0, 18.2, 36.4 and 72.8 ms. Relaxation time constant maps were calculated pixel-by-pixel manner with monoexponential decay function. B_1_ measurements for B_1_ mapping included a hard pulse with power of 625 Hz with increasing durations from 0 to 1.0 ms, Δ = 0.125 ms^43^.

2D fast spin echo (FSE) sequence was used as a readout in all measurements after the preparation pulse. Parameters of FSE were TR = 3000 ms, effective TE = 8.44 ms, averages = 5 with data matrix = 192 × 192 and 1 mm slice thickness. The axial imaging plane was placed in the lower mid level of the heart and imaged as a short-axis view (Table 2).

**Table 2:** Relaxation rate constants detected with RAFF2 and RAFF4 and the corresponding SS values simulation results in the different stretching factors of *P*-packets in RAFF2 pulse and from the original RAFF1-5, where Fn1 and Postn protein concentrations in healthy (original) and myocardial infarction (upregulated) are proportional to water concentration in myocardium. Original RAFF2 method is bolded in the table to be shown as a reference. R refers to relaxation rate constant and SS to steady state formation. τ_c_ = 10·10^− 12^, 9.4 T external magnetic field, gyromagnetic ratio = 42.576 MHz/T for ^1^H in 9.4 T, planck’s constant = 1.054571628·10^− 34^ Js, hydrodynamic radius = 158·10^− 12^ m, permeability of vacuum = 4π·10^− 7^, τ_ex_ = 0.001 Hz, off-resonance for both pools were set to be Δ_A_ = 2·π ·P_B_·500 rad/s and be Δ_B_ = 2·π ·P_A_·500 rad/s, where P_A_=(c(Fn1)/c(water)) or P_A_=(c(Postn)/c(water)) and P_B_=1-P_A_. P_A_ included healthy concentration values of 1.2·10^− 6^ mol/l for Fn1, 1.1·10^− 9^ mol/l for Postn and 80 mol/l for the water concentration. For the upregulated concentration values, we used 1.2·10^− 5^ mol/l for Fn1 and 1.1 ·10^− 8^ for Postn.

Relaxation method	*R* (original) (s^− 1^)	SS (original) (s)	*R* (upregulated) (s^− 1^)	SS (upregulated) (s)
RAFF2 TL0.6	2.25	0.93	2.25	0.93
RAFF2 TL0.8	3.03	0.83	3.03	0.83
**RAFF2 TL1.0**	**3.83**	**0.69**	**3.83**	**0.69**
RAFF2 TL1.2	4.62	0.55	4.62	0.55
RAFF2 TL1.4	5.42	0.42	5.42	0.42
RAFF2 TL1.6	6.21	0.33	6.21	0.33
RAFF2 TL1.8	7.00	0.26	7.00	0.26
RAFF2 TL2.0	7.80	0.21	7.80	0.21
RAFF1	3.89	0.48	3.89	0.48
RAFF3	3.79	0.84	3.79	0.84
RAFF4	3.73	0.98	3.73	0.98
RAFF5	3.72	0.99	3.72	0.99

### MRI data analysis

MI, remote and healthy areas were assessed using regions of interest (ROIs) analysis. MI and remote ROIs were the first drawn in the original RAFF2 (TL1.0) relaxation map^39^ and used that ROI in all other maps in all MI hearts. For the healthy myocardium, the whole myocardium was selected as one ROI and it was used in all healthy control hearts. The ROIs were then used to collect the mean values in specific regions of the data in relaxation maps. The contrast between remote and sub-chronic MI areas was measured as RRTD. The RRTD was calculated as (T(infarct)-T(remote))/T(remote)·100%, where T represents the relaxation time constant in that specific ROI. Results are given in the form of the mean ± standard deviation. Student’s t-test, One-way ANOVA Dunn’s multiple comparison test and Two-way ANOVA with Bonferroni’s post hoc tests were performed to analyze statistically the results, and *p* < 0.05 was considered statistically significant.

### Proteomics

After the MRI, the hearts were frozen and cut in half to separate ventricles and atriums. Infarcted and healthy ventricular and atrial samples (*n* = 3 per group) were homogenized in Abcam protein lysis buffer (ab193970) using the efficient bead-beating protein extraction method^44^. The solubilized heart proteome was extracted by centrifugation at 18,000 × g for 20 min. The extracted proteins were digested and processed using the filter-aided sample preparation (FASP) protocol as previously described^45,46^. The resulting tryptic peptides were analyzed in full-scan and data-independent acquisition (DIA) modes using high-resolution mass spectrometry (Orbitrap Q Exactive Classic) coupled with an ultra-high-performance liquid chromatography system (Vanquish Flex UHPLC, Thermo Scientific, Bremen, Germany) as previously described^46^. The MS/MS spectral library and peptide retention times were predicted using the UniProt reference proteome database for mouse (UP000000589, updated in April 2022, containing 21,957 protein entries) along with an additional UniProt mouse database comprising 41,543 protein isoform entries. Cysteine residues were set as static modifications, while oxidation of methionine and N-terminal acetylation were designated as variable modifications, with a maximum of two variable modifications per peptide. The predicted MS library was used to search the raw data, applying a 1% false discovery rate (FDR) threshold for both precursor and protein groups and requiring at least one proteotypic peptide per protein (7–30 amino acids in length)^47^. The normalized MaxLFQ quantities were subsequently subjected to downstream comparative statistical analysis using Perseus software^48,49^. Categorical groups were analyzed using the LIMMA test^48^, with multiple testing corrections applied via the false discovery rate (FDR), and statistical significance set at q < 0.05.

### Simulations

Two-pool Bloch-McConnell equations were used to model the effect of Fn1 and Postn proteins on RAFF1-5 and RAFF2 TL0.6-TL2.0^26,29^. The identical pools of the T_1_ and T_2_ were calculated by using the model of dipolar interactions between isolated spins using rotational correlation time τ_c_ = 10·10^− 12^ s, similarly to^26^. Other parameters for the simulations were 9.4 T external magnetic field, gyromagnetic ratio was 42.576 MHz/T for ^1^H in 9.4 T, Planck’s constant was 1.054571628·10^− 34^ Js, hydrodynamic radius was 158·10^− 12^ m^50^, permeability of vacuum was 4π·10^− 7^, τ_ex_ was 0.001 Hz for -OH-group, off-resonance frequency for both pools were set to be Δ_A_ = 2·π ·P_B_·500 rad/s and be Δ_B_ = 2·π ·P_A_·500 rad/s, respectively, where P_A_=(c(Fn1)/c(water)) or P_A_=(c(Postn)/c(water)) and P_B_=1-P_A_. P_A_ included healthy concentration values of 1.2·10^− 6^ mol/l for Fn1 (FN1 protein expression summary - The Human Protein Atlas), of 1.1·10^− 9^ mol/l for Postn^51^ and of 80 mol/l for the water concentration. For the upregulated concentration values, we used 1.2·10^− 5^ mol/l for Fn1 and 1.1 ·10^− 8^ for Postn. The α_1_ was 45° and ω_1_^max^ = 2·π·625 rad/s, which was decreasing as the n in the RAFFn was increasing. The number of points in *PP*_π_^−1^*P*_π_*P*^− 1^^23^ waveform was 128 for original RAFFn and were changed as the TL factor changed. This affects the refocusing pulse duration which is calculated with as follows: *T*_p_=(4·π)/($\:\sqrt{2}$ ·ω_1_^max^). A total of 64 pulses were used in the pulse train to form up to 144 ms long RF irradiation, where time points 0 and 32 points were evenly distributed. Also, magnetizations from both + Z and -Z were used to calculate relaxation rates in the simulations and then the magnetizations were summed up, and SS formation^23,41^ of exponential decay function was taken account to fit the data points by using non-linear least square fitting. Waveforms and refocusing schemes were formed with equations previously derived^23,52^. Simulations were performed in Matlab R2023b by numerically solving Bloch-McConnell equations by ordinary differential equation solver (ode45) time point-by time point fashion.

### Histology

For the two hearts, standard tissue processing and paraffin embedding methods were used. 4 μm thick cross-sections of the hearts were cut and stained with Picro Sirius Red staining. The slides were mounted with Permount (ThermoFisher Scientific), and the sections were photographed using a Nikon Eclipse microscope with a Ds-Ri2 camera (Nikon Instruments Europe BV).

## Data Availability

The datasets used and/or analyzed during the current study are available from the corresponding author on reasonable request.
